# F-18 Fluoro-2-Deoxyglucose Positron Emission Tomography (PET)/Computed Tomography (CT) Imaging in Melanoma: Normal Variants, Pitfalls, and Artifacts

**DOI:** 10.3389/fnume.2022.835404

**Published:** 2022-03-08

**Authors:** Jaleelat I. Momodu, Mboyo Di Tamba Vangu

**Affiliations:** Department of Nuclear Medicine and Molecular Imaging, Charlotte Maxeke Johannesburg Academic Hospital, University of the Witwatersrand, Johannesburg, South Africa

**Keywords:** [18F]FDG, FDG, PET, PET/CT, melanoma, variants, pitfalls, artifacts

## Abstract

Multimodality imaging has revolutionized diagnostic imaging for several oncologic pathologies including melanoma. Although F-18 fluoro-2-deoxyglucose positron emission tomography/ computed tomography [18F]FDG PET/CT has a high sensitivity in stage III and IV melanoma, several normal variants, and imaging pitfalls may result in falsely increased or reduced tracer uptake that may negatively impact diagnostic accuracy. In addition to normal physiologic tracer uptake, differences in the biological and molecular characteristics of different types of melanoma are also responsible for pitfalls. For instance, [18F]FDG PET/CT has a low sensitivity for detecting brain metastases due to normal physiologic [18F]FDG uptake in brain tissue while hepatic metastases from cutaneous melanoma are more [18F]FDG-avid than hepatic metastases from uveal melanoma. With the introduction of immunotherapies for melanoma, treatment response assessment using [18F]FDG PET/CT has a reduced specificity. This is due to hypermetabolic immune-related adverse effects such as hepatitis, dermatitis, and colitis resulting in false-positive uptake. In addition, immune therapy-induced initial increase in tumor uptake followed by disease response (pseudo-progression) is a cause of false-positive scan interpretation. Specific technical artifacts impact disease detection in [18F]FDG PET/CT melanoma imaging. The identification of small metastatic lymph nodes and lung nodules may be limited by the resolution of the PET/CT camera (partial volume effect). Computed tomography (CT) attenuation correction results in less apparent skin and subcutaneous lesions. Pictorial illustrations will be central to this paper for the description of these normal variants, imaging artifacts, and pitfalls. It is critical for the imaging specialist to have a clear understanding of these potential limitations of ^18^F-FDG PET/CT imaging in individuals who are referred with melanoma.

## Introduction

Worldwide incidence of the malignant transformation of pigment producing cells has been rising steadily (1). The most common form of melanoma arises from melanocytes in the skin (cutaneous melanoma) and is also the most fatal skin cancer (2). Melanoma can also arise from extra-cutaneous pigment-producing cells in the eyes, gastrointestinal tract, genitalia, sinuses, and meninges (2, 3). The American Joint Committee on Cancer (AJCC) 8th edition categorizes melanoma by the TNM staging which defines localized disease (stage I and II), node positive disease (stage III) and advanced or metastatic disease (stage IV) (4, 5). The key clinical and pathological features that impact melanoma staging are tumor thickness (known as the Breslow depth), ulceration, rate of mitosis, presence of microsatellites nodes and in-transit lesions, tumor status of regional lymph nodes and presence of distant metastasis (2). Multimodality imaging has revolutionized diagnostic imaging for several oncologic pathologies, including melanoma.

The clinical relevance of hybrid [18F]FDG PET/CT imaging is in the staging and re-staging of high risk melanoma (AJCC Stages III and IV), where there is good sensitivity and specificity for identifying additional disease sites (5, 6). The valuable performance of [18F]FDG PET/CT is well-established in N- and M- staging of cutaneous melanoma at different phases of diagnosis that include primary staging, response assessment post-chemotherapy, staging of suspected recurrence and in determining prognosis (7). Other indications for [18F]FDG PET/CT include guiding treatment (surgery, radiotherapy) and more recently, to assess and predict response to newer therapies (3). Although [18F]FDG PET/CT also plays a significant role in response assessment post-immunotherapy, specificity may be impacted by false positive findings due to normal patient physiology, tracer kinetics, treatment response, and inflammatory adverse effects.

## Normal Variants and Pitfalls in Staging and Disease Recurrence

Due to the low risk of distant metastases and PET camera resolution limitations for detecting microscopic nodal disease, [18F]FDG PET/CT plays a limited role in staging early melanoma (AJCC stages I and II) (5, 6). Conversely, [18F]FDG PET/CT has value in the initial staging of advanced cutaneous melanoma (AJCC stages III and IV), where it demonstrates a high accuracy in the detection of lymph node, soft tissue, and visceral metastases (8). Hybrid [18F]FDG PET/CT shows superior accuracy to conventional imaging for identifying loco-regional and distant metastases and is able to provide critical information to guide management decisions for recurrent disease in cutaneous melanoma (9).

Normal variants on [18F]FDG PET/CT imaging for staging high risk melanoma are attributed to normal tracer uptake in tissues or benign conditions not related to malignancy. Since the acquisition of PET in melanoma often requires imaging the whole body from brain to toes, normal variants may be found at any anatomical region of the body.

**Figure 1:**
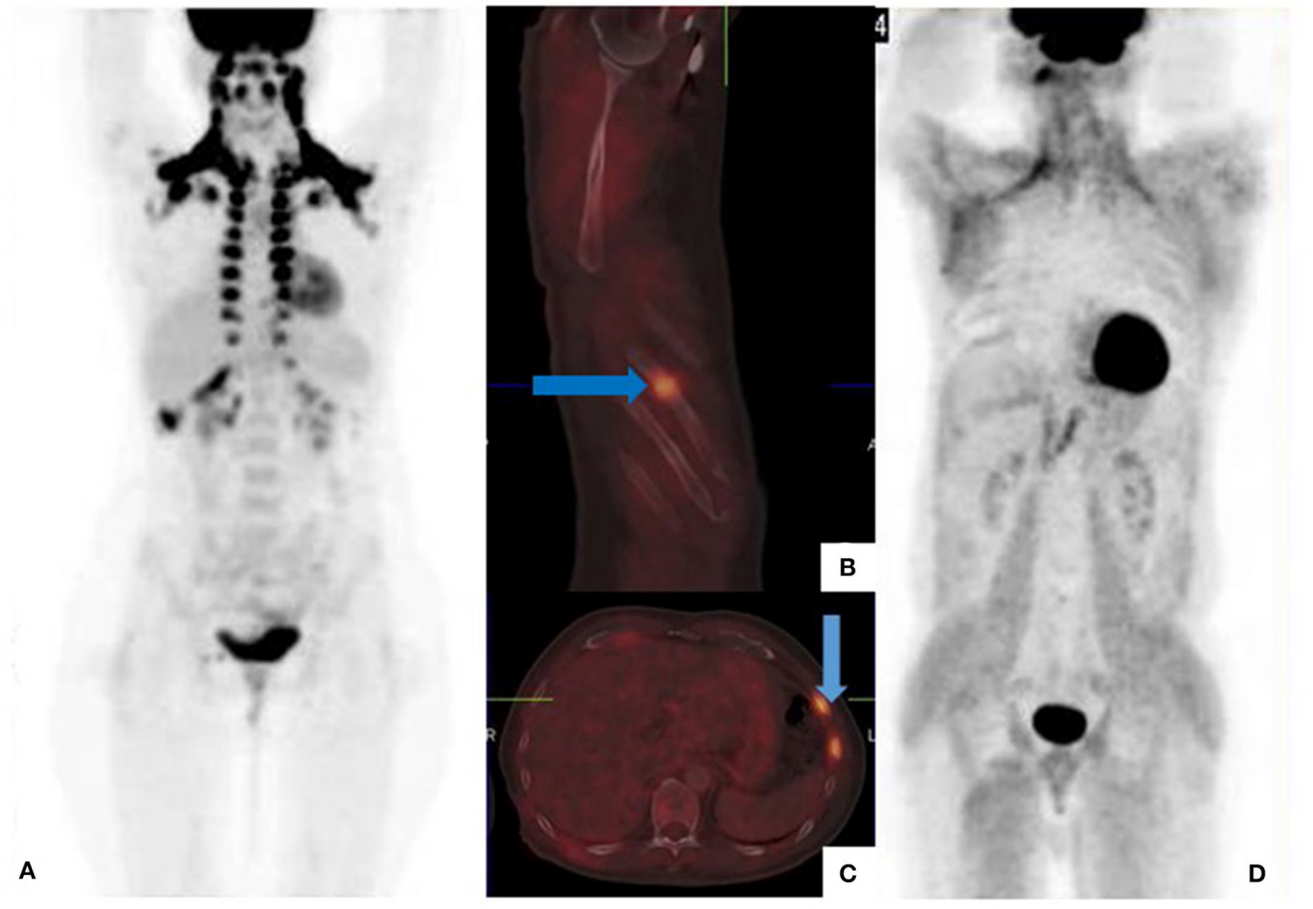
[18F]FDG PET/CT for staging malignant melanoma of the upper lip **(A)** and back **(B–D)**. Maximum intensity projection (MIP) shows symmetrical brown fat uptake in the cervical, supraclavicular, mediastinal, and paraspinal regions **(A)**. Fused sagittal **(B)** and trans-axial **(C)** images show increased tracer uptake in the 6th, 7th, and 8th anterior-lateral consecutive ribs with callus formation noted on CT. These findings represent fractures from previous trauma and not metastatic disease. Generalized muscle uptake **(D)** was noted in a patient who inadvertently injected his insulin on the morning of the scan.

Brown adipose tissue (BAT) or brown fat is involved in thermogenesis and creates heat through glucose metabolism (10). BAT activation is usually triggered by low temperatures and occurs more frequently in females, younger and thinner patients. On [18F]FDG PET/CT, BAT activation presents as symmetrical increased uptake in the cervical, supraclavicular, and paravertebral regions (10) (Figure 1A). Brown fat uptake can interfere with scan interpretation, particularly when the primary disease site is in the head and neck or trunk. Measures to minimize brown fat activation include wearing warm clothing, use of blankets as well as administration of beta-blockers or benzodiazepines (11).

Recent bone fractures, healing bone fractures, inflammatory and degenerative bone disorders demonstrate intense [18F]FDG uptake and can mimic skeletal metastases (12). The pattern of skeletal uptake and clinical history will guide image interpretation (Figure 1B).

Imaging pitfalls are related to a number of factors including patient preparation, lesion characteristics as well as organ and primary site dependent variability in [18F]FDG uptake. Pitfalls result in false positives or false negatives which can adversely impact scan sensitivity and specificity.

To minimize competitive inhibition between serum glucose (in the hyperglycemic state) and [18F]FDG, patients are required to fast for a minimum of 4–6 h prior to the [18F]FDG PET/CT study (13). In the non-fasting state, endogenous insulin production is stimulated which results in [18F]FDG deposition in insulin sensitive tissues such as fat and skeletal muscle (Figure 1C). Consequently, there is a relative reduction in [18F]FDG tumor uptake, leading to masking of tumor lesions and false negative assessments. Similar findings occur following exogenous insulin administration prior to the PET scan (Figure 1D).

**Figure 2:**
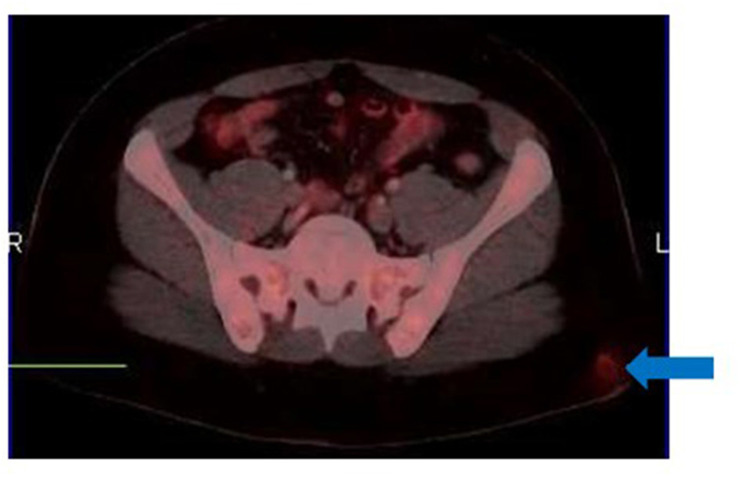
[18F]FDG PET/CT for suspected recurrent cutaneous melanoma. Fused trans-axial image shows a focus of uptake in the left gluteal region (arrow) mimicking subcutaneous involvement. There was no associated CT change. This focus corresponds to recent intramuscular injection site as confirmed from the clinical history.

Voluntary muscle contraction during the uptake period increases skeletal muscle uptake, and limits study accuracy. Patients should avoid physical exercise for at least 6 h prior to the [18F]FDG PET/CT study (13). In addition, focal tracer uptake at recent injection sites may be mistaken for subcutaneous metastases (Figure 2).

[18F]FDG PET/CT has limited sensitivity in detecting small sized primary lesions and micro-metastatic disease below the resolution of the PET scanner which ranges from 5 to 6 mm (7).

**Figure 3:**
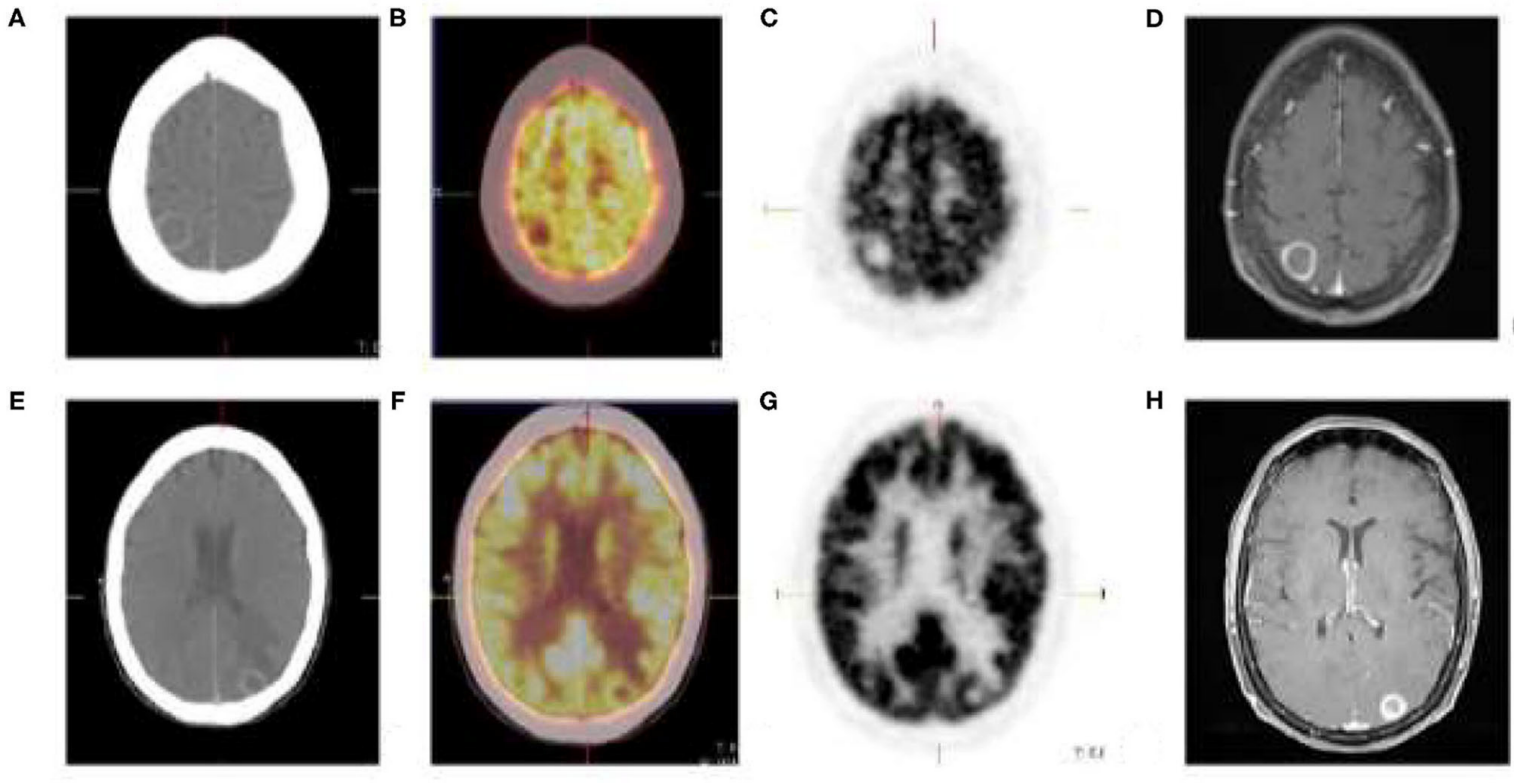
Cutaneous melanoma of the right thigh with known inguinal nodal metastases. [18F]FDG PET/CT shows rim enhancing parietal **(A)** and occipital **(E)** brain lesions with no discernable metabolic activity **(B,C,F,G)**. Brain metastases was confirmed on MRI **(D,H)**.

Advanced stage melanoma is associated with a high rate of brain metastasis, and consequently poor prognosis (12, 14). At initial presentation, the incidence of brain metastases is ~20% with up to 50% of patients with stage IV melanoma developing brain metastases during the course of their disease (15). The sensitivity of [18F]FDG PET/CT for detecting brain metastases is poor due to high physiologic [18F]FDG metabolism in normal brain parenchyma, resulting in reduced target to background ratio (Figure 3). Another pitfall is the limited detection of small sized lesions due to spatial resolution limitations of the camera system.

**Figure 4:**
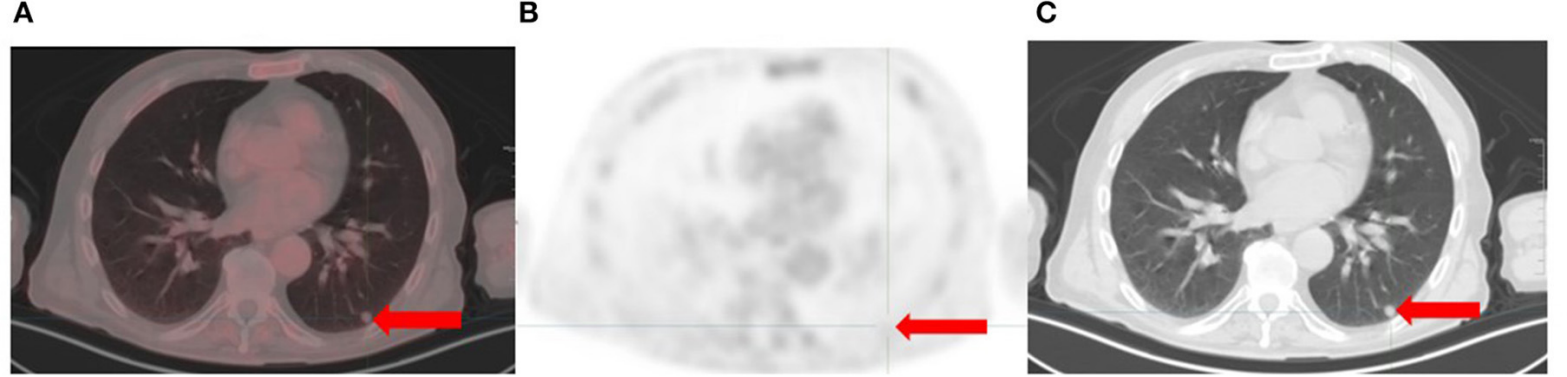
Recurrent melanoma with a 9 mm left lung nodule (arrows). There is no metabolism on the [18F]FDG PET/CT trans-axial fused **(A)** and PET only **(B)** images. CT features are suspicious for malignancy **(C)**.

Pulmonary metastases is reported in up to 40% of melanoma cases and is associated with a short median overall survival (16). Factors that impact detection of lung metastases on [18F]FDG PET/CT include lesion size, spatial resolution of the PET camera and involuntary chest wall motion which can result in breathing artifacts (12). [18F]FDG PET/CT has a reduced sensitivity for detecting small metastatic lung lesions due to partial volume effect, resulting in underestimation of tracer uptake (Figure 4). In an analysis of 181 pulmonary metastases, [18F]FDG PET/CT sensitivity for detection was only 7.9% for lesions 4–5 mm compared to 100% for lesions 12–14 mm (17).

**Figure 5:**
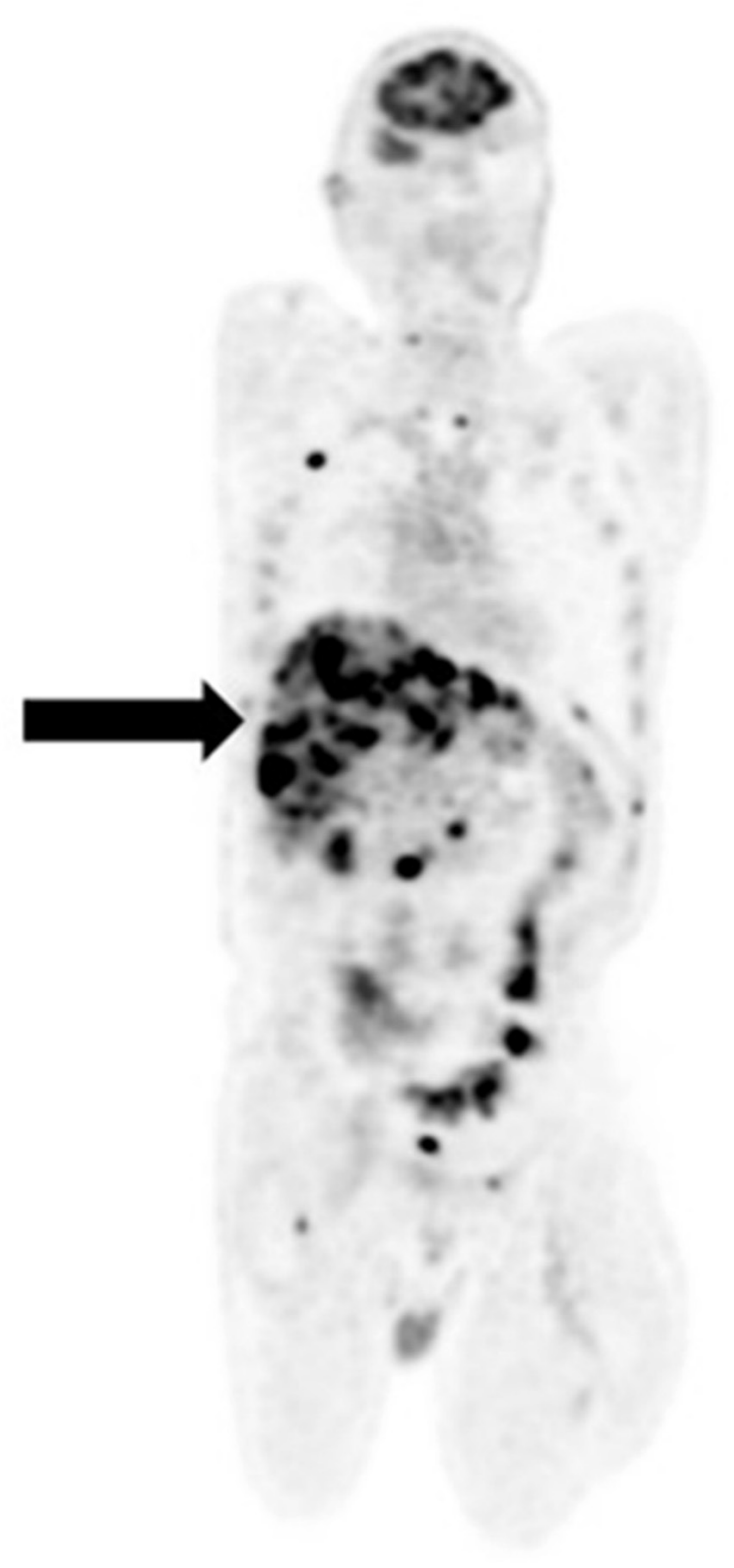
Recurrent cutaneous melanoma with widespread metastases. [18F]FDG PET/CT coronal image shows intense avidity of liver lesions (arrow).

[18F]FDG PET/CT imaging for liver metastases from melanoma is associated with a number of pitfalls. First, [18F]FDG PET has a low sensitivity for detecting liver metastases from most types of melanoma and is associated high false negative rates (9). Secondly, hepatic adenoma is a potential source of false positive imaging in suspected liver metastases as both are hypervascular and may demonstrate hypermetabolism on [18F]FDG PET (18). Furthermore, liver metastases from different types of melanoma demonstrate different levels of [18F]FDG avidity. Whereas, liver metastases from cutaneous melanoma are reliably [18F]FDG -avid (Figure 5), those from uveal melanoma show low [18F]FDG avidity resulting in false negative assessments (3). The reasons for this disparity are not clearly understood.

**Figure 6:**
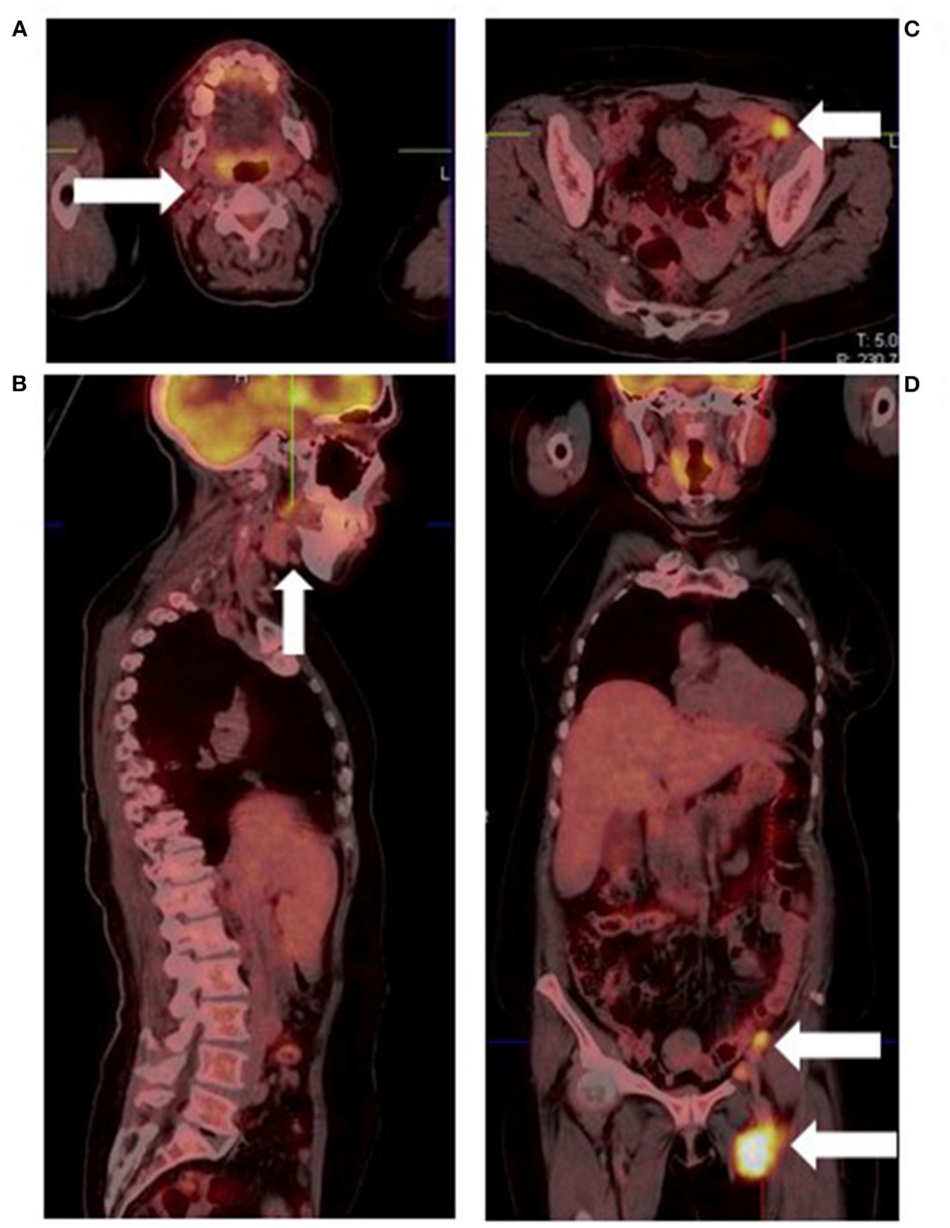
Malignant melanoma of the left heel in a known retroviral disease patient. Fused [18F]FDG PET/CT images show cervical **(A)**, submandibular **(B)**, and abdominopelvic lymphadenopathy **(C,D)**. Based on their proximity to the primary site and intense hypermetabolism, the abdominopelvic lymph nodes represent metastatic sites. Conversely, the low grade cervical and submandibular lymph nodes most likely represent retroviral disease.

Imaging lymph node metastases in melanoma with [18F]FDG PET/CT presents a number of challenges. First, uptake is non-specific as infective, inflammatory and other malignant conditions also demonstrate [18F]FDG nodal uptake, resulting in false positives (Figure 6). Secondly, detection of small, microscopic nodal disease is compromised by the limited special resolution of PET.

**Figure 7:**
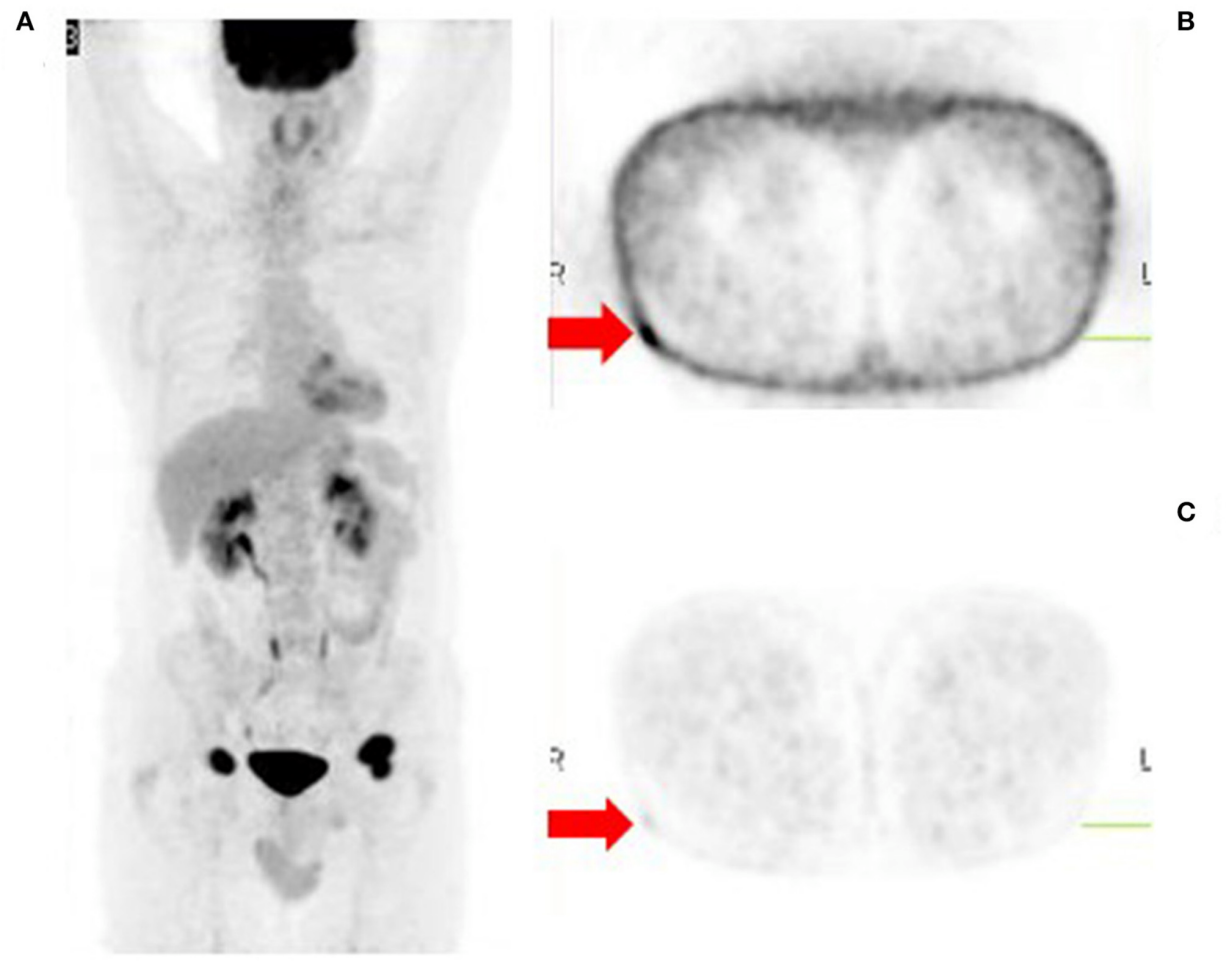
Metastatic malignant melanoma diagnosed from biopsy of inguinal lymph nodes. [18F]FDG PET/CT was done to identify the primary site of disease. The MIP image **(A)** shows bilateral inguinal lymphadenopathy and no other sites of metabolically active disease. However, close inspection of the non-attenuation corrected images **(B)** reveals a small subcutaneous focus in the lateral aspect of the right upper thigh, the likely site of primary disease. This focus is poorly visualized on the corresponding corrected images **(C)**.

Assessment of skin and subcutaneous lesions on [18F]FDG PET/CT requires caution to avoid interpretation errors. Intense cutaneous activity on non-attenuation corrected images due to close proximity of the skin to the PET camera detector may mask malignant skin lesions, resulting in false negative assessments. On the other hand, peripheral subcutaneous lesions may be better appreciated on non-attenuation corrected images (Figure 7) due to the lack of attenuation relative to central structures (9). Thus, when evaluating the skin and soft tissues for local recurrence or cutaneous metastases, it is important to review both corrected and non-attenuation corrected images.

**Figure 8:**
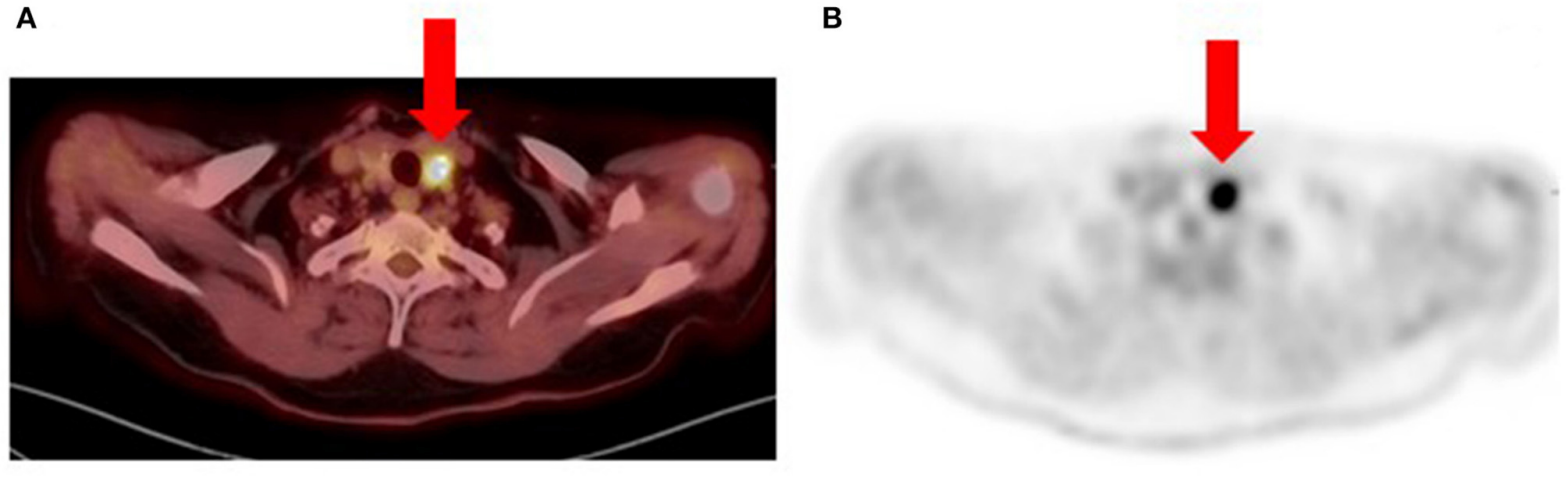
Patient with recurrent metastatic cutaneous melanoma had an [18F]FDG PET/CT for re-staging. Fused **(A)** and PET-only **(B)** trans-axial images show a bulky nodular thyroid gland with multiple calcifications. The largest nodule is in the left lobe (arrows) with intense hyper-metabolism (SUV_max_ 20.9) and associated calcifications. Fine needle aspiration cytology of the thyroid nodules was suspicious for papillary thyroid carcinoma (Bethesda Category V). Histology from total thyroidectomy specimen confirmed the diagnosis of a secondary malignancy and patient subsequently received radioactive iodine therapy.

Metastatic melanoma to the thyroid gland is rare, occurring in only 4% of cases as determined in a meta-analysis by Chung et al. (19). Therefore, focal hypermetabolism in the thyroid gland on [18F]FDG PET/CT imaging for newly diagnosed or recurrent melanoma may represent a secondary malignancy rather than metastatic disease (Figure 8).

## Normal Variants and Pitfalls in Treatment Response Assessment

The role of hybrid imaging is established in the assessment of treatment response following conventional modalities (surgery, chemotherapy, and radiation therapy) as well as immunotherapy (20). Although [18F]FDG PET/CT has the advantage of being able to differentiate treatment related changes such as radiation necrosis from relapse, there are a number of normal variants and pitfalls that can impact reporting.

**Figure 9:**
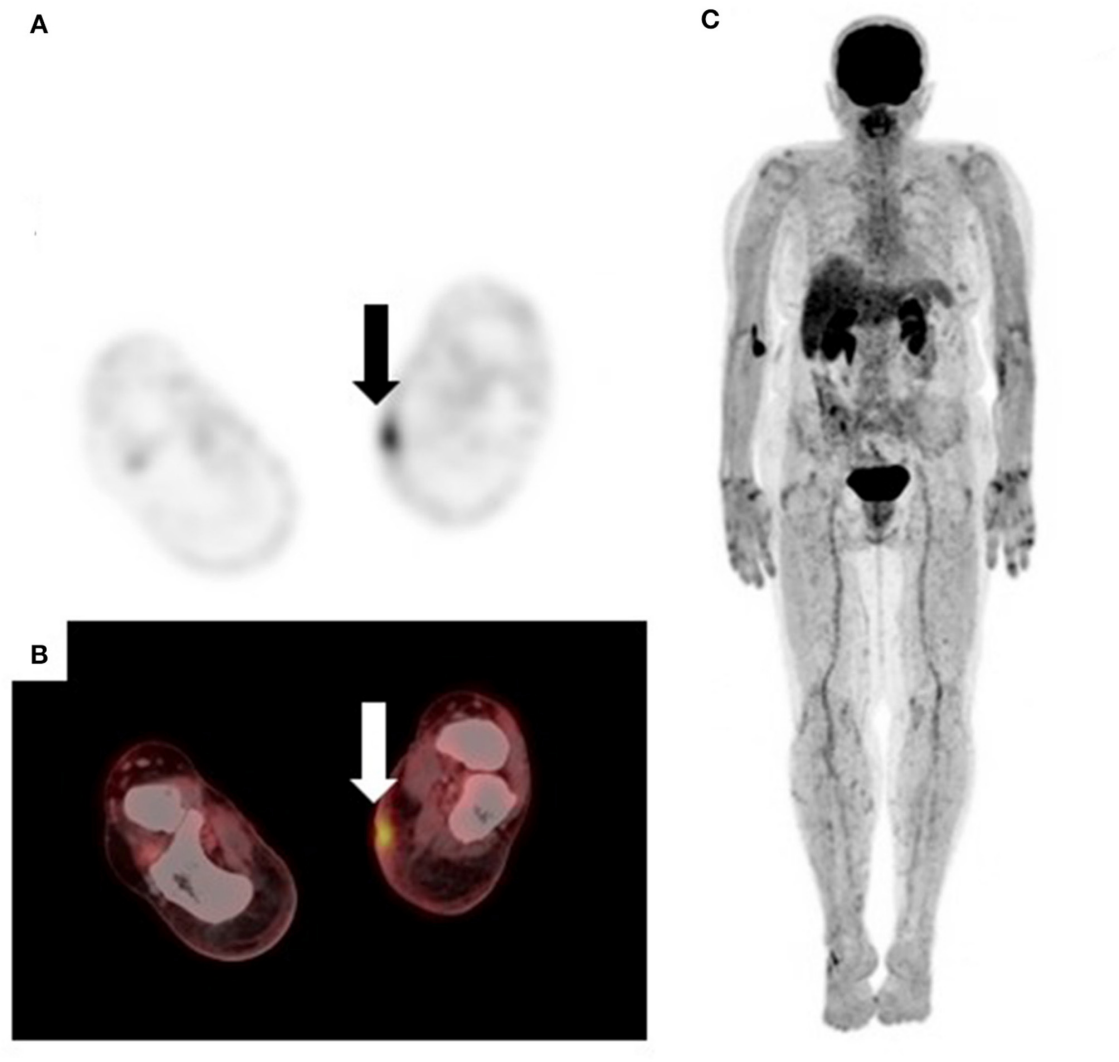
[18F]FDG PET-only **(A)** and fused PET/CT trans-axial **(B)** images show increased diffuse subcutaneous uptake (SUV_max_ 6.03) in a case of invasive malignant melanoma of the left foot. Patient recently had a wider excision after initial excision showed disease extension to the resection margins. Maximum intensity projection **(C)** of a left forearm melanoma post-excision 2 months prior to PET scan shows linear subcutaneous uptake in the excision site (SUV_max_ 3.8). Focal uptake in the right cubital fossa corresponds to the site of radiotracer injection.

Post-inflammatory changes occurring around the site of previous surgical resection may mimic disease recurrence, a source of false positive imaging (Figure 9). Increased cellular activity mediating inflammation and wound repair causes increased [18F]FDG uptake. Compared to recurrence, post-surgical changes tend to have low grade uptake and a diffuse pattern (12).

**Figure 10:**
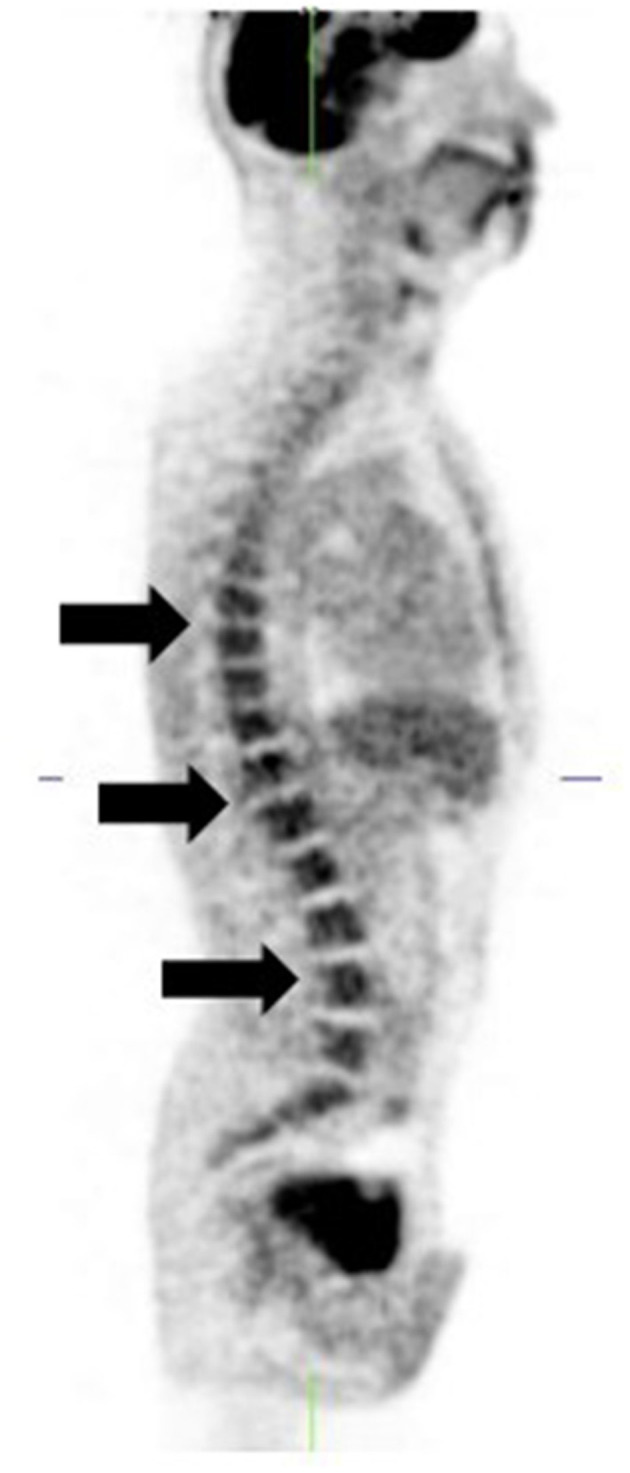
[18F]FDG PET-only sagittal image shows diffusely increased bone marrow uptake (arrows). Patient is a stage 4 melanoma 6 weeks post chemotherapy.

Recent treatment with chemotherapy or granulocyte-colony stimulating factor (G-CSF) results in diffusely increased bone marrow uptake which may be mistaken for metastases (12) (Figure 10). Also, cessation of therapy with the myelosuppressant, interferon alpha, may produce a compensatory rebound marrow hyper-stimulation with increased [18F]FDG uptake (21) which can mimic metastatic disease.

Rebound splenic hyper-stimulation syndrome following chemotherapy is characterized by diffusely increased [18F]FDG uptake in the splenic parenchyma and bone marrow (12). This can be differentiated from splenic metastases which presents as focal area(s) of hyper-metabolism in the spleen.

The role of immunotherapy in the management of metastatic melanoma has evolved over the years and currently involves immune system activation via checkpoint blockade (22). Immunotherapy has the ability to destroy both grossly visible disease as well as micro-metastases, providing significant benefit over other conventional treatment modalities (22). The Food and Drug Administration (FDA)-approved immunotherapies for metastatic cutaneous melanoma include: cytokines, high dose interleukin 2 (IL-2), immune checkpoint inhibitors targeting cytotoxic T lymphocyte antigen 4 (CTLA-4)- ipilimumab and programmed cell death 1 (PD-1)- nivolumab and pembrolizumab (23, 24). Several studies have demonstrated the value of [18F]FDG PET/CT as a predictive biomarker of response and for post-treatment response assessment in patients with melanoma receiving immunotherapy (22, 25). Since immunotherapies work by reactivating the immune system, they are associated with toxicity profiles also described as immune related adverse effects (irAEs). These adverse effects are associated with lymphocytic infiltration and inflammation in various tissues and organ systems resulting in thyroiditis, pneumonitis, gastritis, colitis, hepatitis, adrenalitis, pancreatitis, and reactive lymphadenopathy (22, 23). On [18F]FDG PET/CT, there is diffusely increased metabolic activity in the affected organ which may be mistaken for malignant disease.

**Figure 11:**
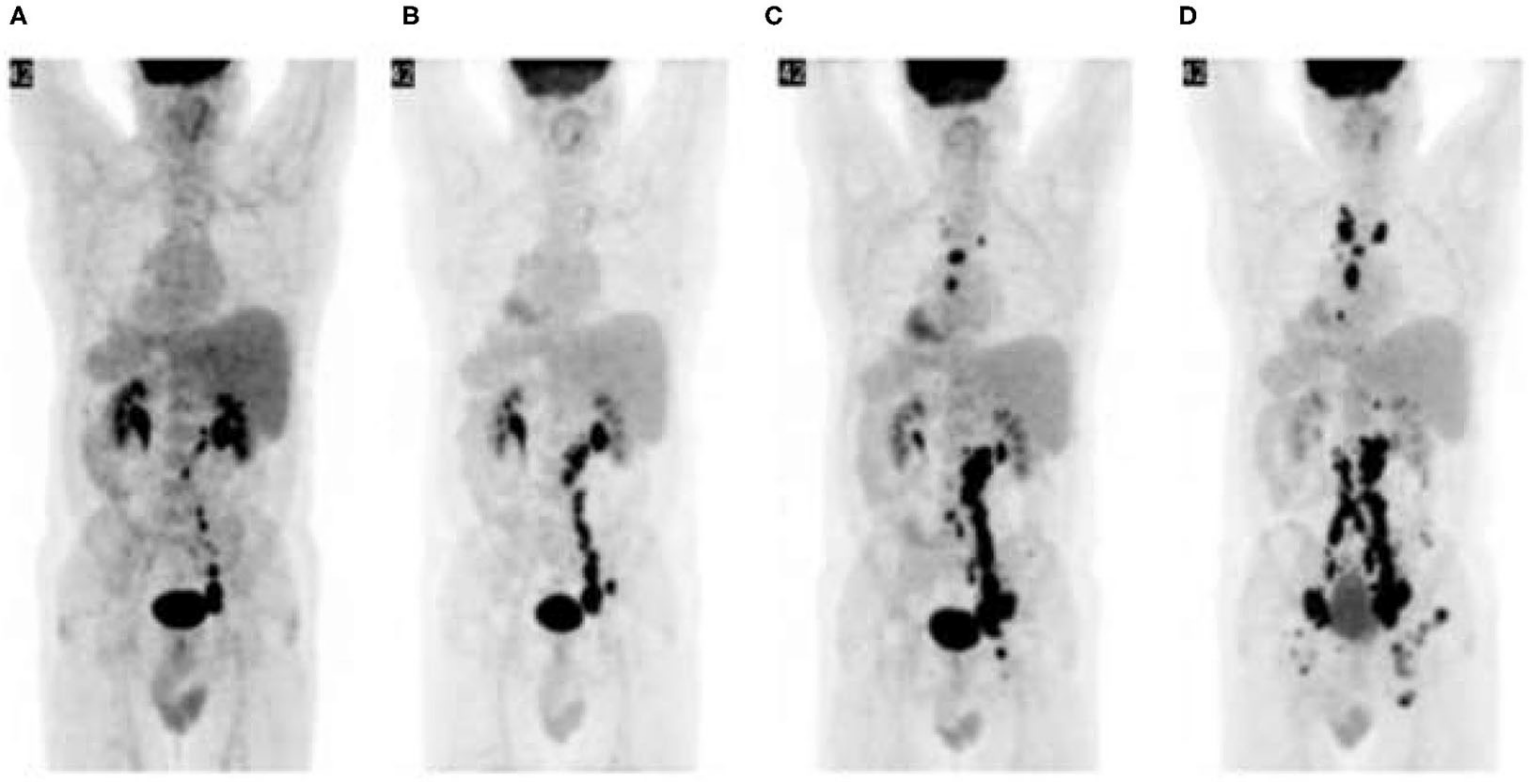
Hyper-progression: Malignant melanoma with failed response to chemotherapy. [18F]FDG PET/CT prior to initiation of ipilimumab shows abdominopelvic lymphadenopathy **(A)**. Repeat PET/CT **(B)** after two cycles of ipilimumab shows disease progression which is persistent after subsequent treatment cycles **(C,D)**. Immunotherapy was ultimately terminated and other treatment options explored.

Immunotherapy is also associated with some distinct treatment response patterns. Pseudo-progression refers to therapy-induced inflammation mimicking progressive disease. It is characterized by therapy response preceded by apparent disease progression (worsening [18F]FDG PET/CT scan findings) (25). Pseudo-progression is a retrospective diagnosis and implies a favorable prognosis. “Hyper-progression” on the other hand, refers to acceleration of tumor growth kinetics (25) and is characterized by worsening clinical and [18F]FDG PET/CT scan findings (Figure 11). It implies true disease progression and indicates a worse prognosis.

## Artifacts

**Figure 12:**
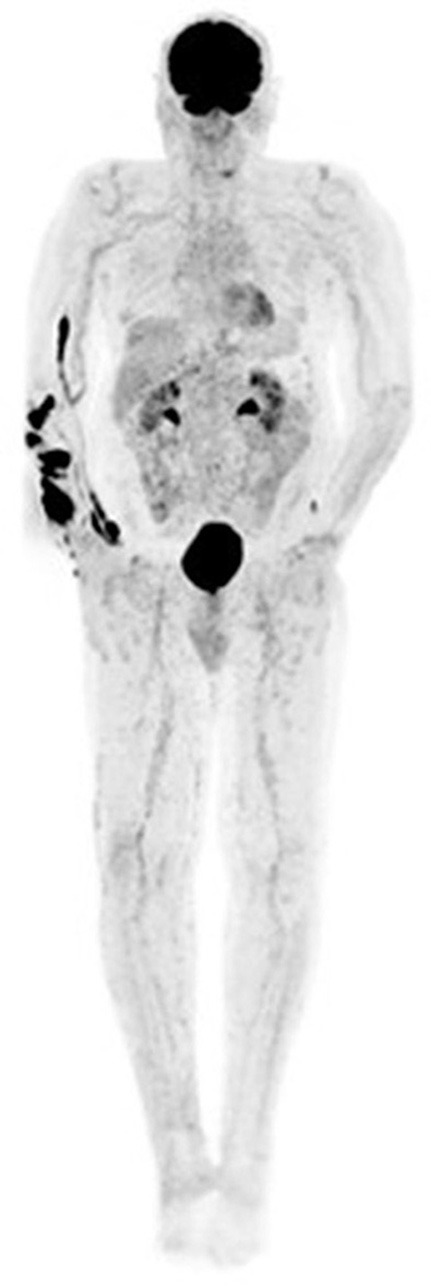
MIP image showing tracer extravasation following multiple injection attempts in the right forearm. This intense areas of uptake can mask pathologic cutaneous, subcutaneous and nodal foci, reducing scan sensitivity.

**Figure 13:**
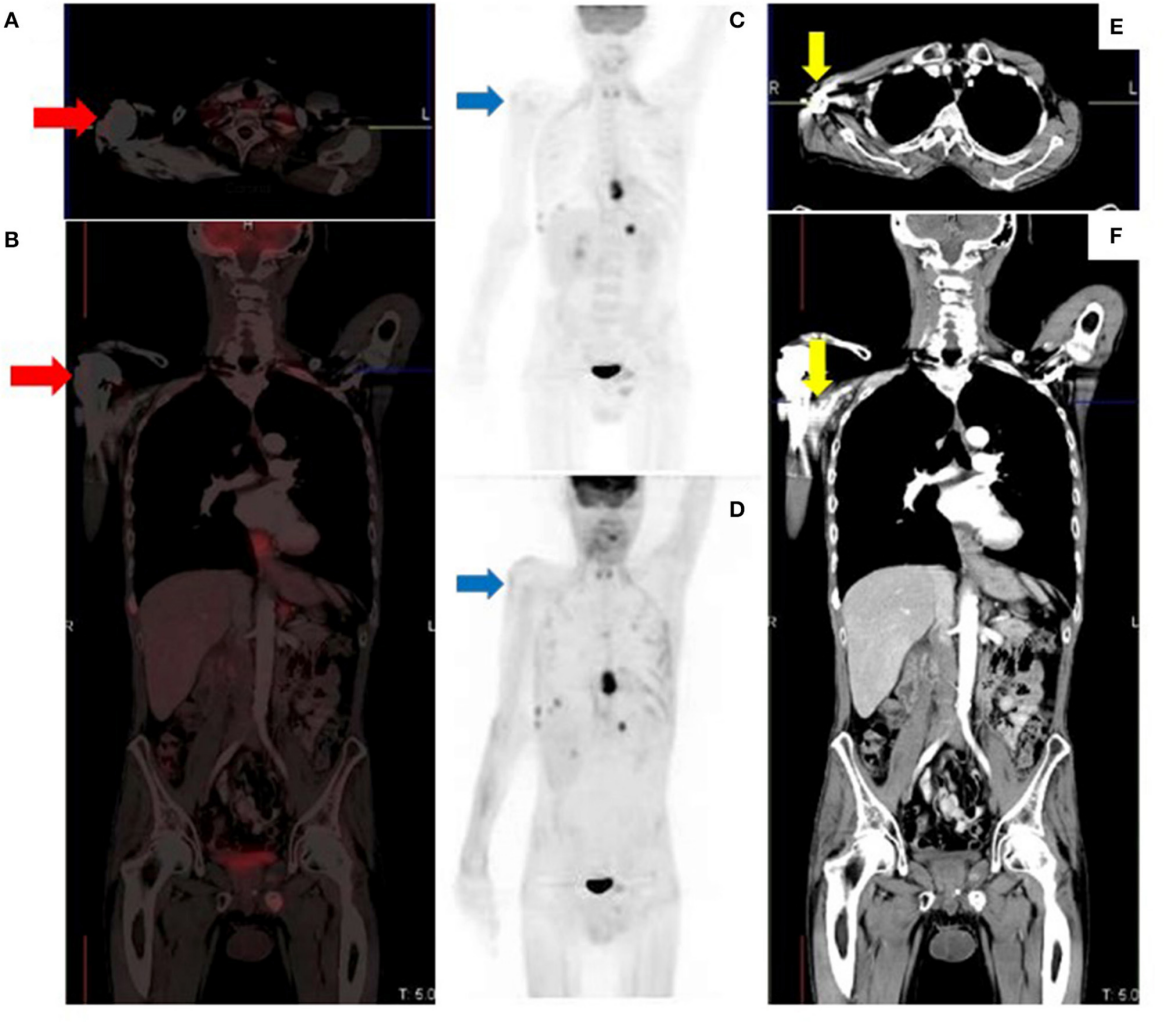
Metallic prosthetic right shoulder implant with overcorrection artifact resulting in mildly increased uptake (red arrows) on the fused **(A,B)** and attenuation corrected **(C)** images. There is no demonstrable peri-prosthetic uptake on the non-attenuation corrected image **(D)**. The implant produces streak artifacts (yellow arrows) best appreciated on the CT images **(E,F)**.

**Figure 14:**
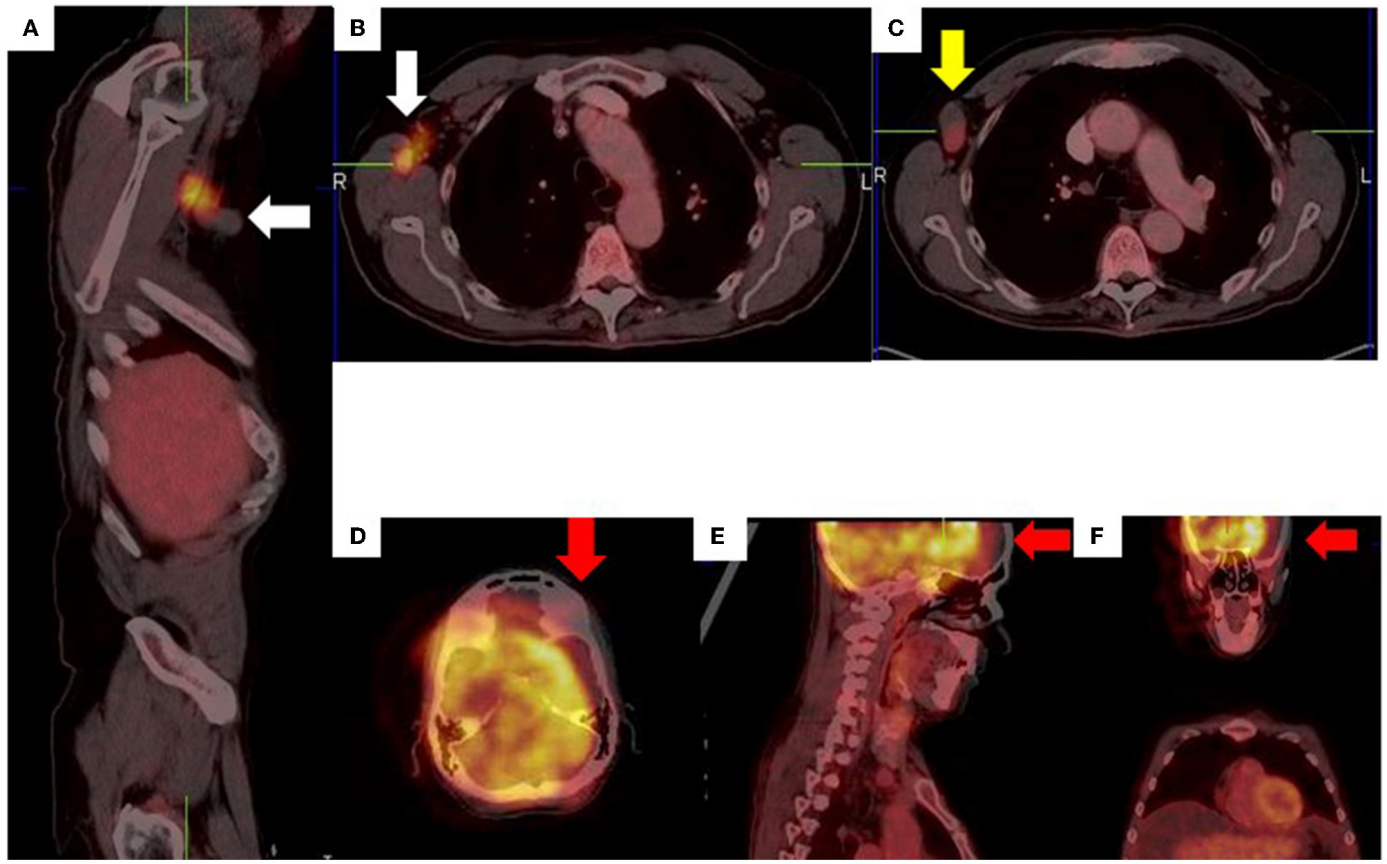
Right axillary lymph node misregistration artifact due to limb motion **(A–C)**. Intense uptake in the right axilla does not conform to a specific anatomical structure (white arrows) while the axillary lymph node demonstrates low grade uptake (yellow arrow). Misregistration between PET and CT images **(D–F)** due to voluntary head motion.

**Figure 15:**
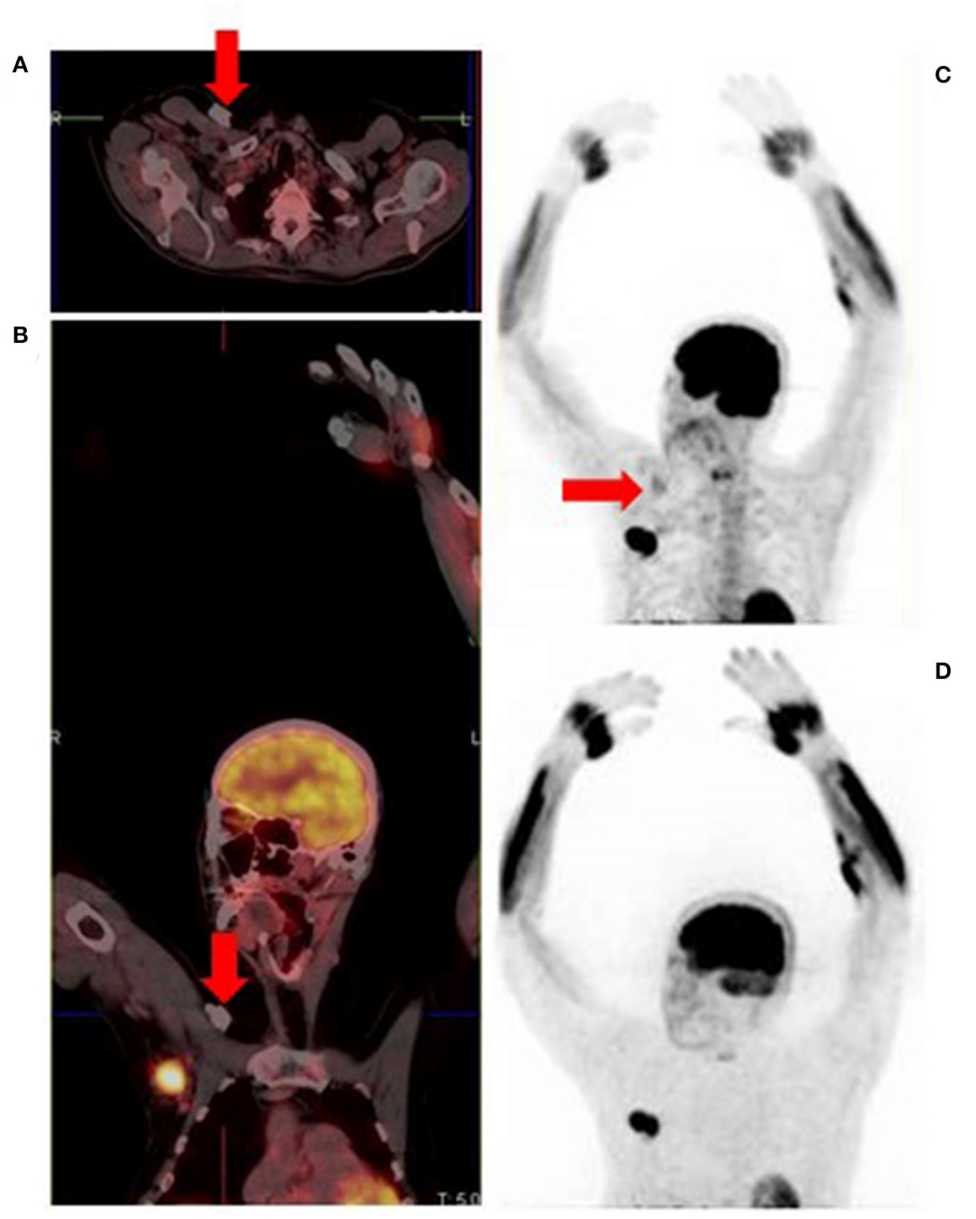
Attenuation correction artifact of a chemotherapy port **(A,B)** results in cutaneous uptake on the corrected image **(C)** which can be mistaken for cutaneous or subcutaneous metastatic disease. There is no uptake on the corresponding non-attenuation corrected image **(D)**.

Knowledge of imaging artifacts in hybrid PET/CT imaging is important as they are potential sources of error in reporting. Imaging artifacts in [18F]FDG PET/CT for melanoma are not different from those described in other oncologic pathologies. Therefore, this section will focus on images and their descriptions without including a long narrative, an avoidable repetition on the topic (Figures 12–15).

## Conclusion

Hybrid imaging with [18F]FDG PET/CT is an essential tool in the management of patients with melanoma. To optimize image interpretation, an understanding of the normal variants, pitfalls and potential artifacts is essential.

## Author Contributions

JM primary writer, literature review, and data collection. MV writing and reviewing the manuscript. All authors contributed to the article and approved the submitted version.

## Conflict of Interest

The authors declare that the research was conducted in the absence of any commercial or financial relationships that could be construed as a potential conflict of interest.

## Publisher's Note

All claims expressed in this article are solely those of the authors and do not necessarily represent those of their affiliated organizations, or those of the publisher, the editors and the reviewers. Any product that may be evaluated in this article, or claim that may be made by its manufacturer, is not guaranteed or endorsed by the publisher.

## References

[1] Schadendorf D van Akkooi ACJ Berking C Griewank KG Gutzmer R Hauschild A. Melanoma. Lancet. (2018) 392:971–84. 10.1016/S0140-6736(18)31559-9 30238891

[2] Jenkins RW Fisher DE. Treatment of advanced melanoma in 2020 and beyond. J Invest Dermatol. (2021) 141:23–31. 10.1016/j.jid.2020.03.943 32268150 PMC7541692

[3] Aide N Iravani A Prigent K Kottler D Alipour R Hicks RJ. PET/CT variants and pitfalls in malignant melanoma. Cancer Imaging. (2022) 22:3. 10.1186/s40644-021-00440-4 34983677 PMC8724662

[4] Gershenwald JE Scolyer RA Hess KR Sondak VK Long GV Ross MI. Melanoma staging: evidence-based changes in the American Joint Committee on Cancer eighth edition cancer staging manual. CA Cancer J Clin. (2017) 67:472–92. 10.3322/caac.21409 29028110 PMC5978683

[5] Van de Wiele C Juanito G Vander BK Lawal I Sathekge M Maes A. Practical considerations when interpreting FDG PET/CT imaging for staging and treatment response assessment in Melanoma patients. Semin Nucl Med. (2021) 51:544–53. 10.1053/j.semnuclmed.2021.06.010 34246450

[6] Schröer-Günther MA Wolff RF Westwood ME Scheibler FJ Schürmann C Baumert BG. F-18-fluoro-2-deoxyglucose positron emission tomography (PET) and PET/computed tomography imaging in primary staging of patients with malignant melanoma: a systematic review. Syst Rev. (2012) 1:62. 10.1186/2046-4053-1-62 23237499 PMC3536719

[7] Perng P Marcus C Subramaniam RM. 18F-FDG PET/CT and melanoma: staging, immune modulation and mutation-targeted therapy assessment, and prognosis. Am J Roentgenol. (2015) 205:259–70. 10.2214/AJR.14.13575 26204273

[8] Bisschop C de Heer EC Brouwers AH Hospers GAP Jalving M. Rational use of 18F-FDG PET/CT in patients with advanced cutaneous melanoma: a systematic review. Crit Rev Oncol Hematol. (2020) 153:103044. 10.1016/j.critrevonc.2020.103044 32673997

[9] Lin E Alavi A. PET and PET/CT- a clinical guide. 2nd Edn. Angewandte Chemie International Edition, 6: 951–952. Thieme Medical Publishers, Inc (2005). p. 1–275.31058910

[10] Steinberg JD Vogel W Vegt E. Factors influencing brown fat activation in FDG PET/CT: a retrospective analysis of 15,0001 cases. Br J Radiol. (2017) 90:20170093. 10.1259/bjr.20170093 28590773 PMC5594988

[11] Cohade C. Altered biodistribution on FDG-PET with emphasis on brown fat and insulin effect. Semin Nucl Med. (2010) 40:283–93. 10.1053/j.semnuclmed.2010.02.001 20513450

[12] Bourgeois AC Chang TT Fish LM Bradley YC. Positron emission tomography/computed tomography in melanoma. Radiol Clin North Am. (2013) 51:865–79. 10.1016/j.rcl.2013.06.004 24010910

[13] Boellaard R Delgado-Bolton R Oyen WJG Giammarile F Tatsch K Eschner W. FDG PET/CT: EANM procedure guidelines for tumour imaging: version 2.0. Eur J Nucl Med Mol Imaging. (2015) 42:328–54. 10.1007/s00259-014-2961-x 25452219 PMC4315529

[14] Galldiks N Langen KJ Albert NL Chamberlain M Soffietti R Kim MM. PET imaging in patients with brain metastasis—report of the RANO/PET group. Neuro Oncol. (2019) 21:584–95. 10.1093/neuonc/noz003 30615138 PMC6502495

[15] Davies MA Liu P McIntyre S Kim KB Papadopoulos N Hwu WJ. Prognostic factors for survival in melanoma patients with brain metastases. Cancer. (2011) 117:1687–96. 10.1002/cncr.25634 20960525

[16] Stadelmann SA Blüthgen C Milanese G Nguyen-Kim TDL Maul JT Dummer R. Lung nodules in melanoma patients: morphologic criteria to differentiate non-metastatic and metastatic lesions. Diagnostics. (2021) 11:837. 10.3390/diagnostics11050837 34066913 PMC8148527

[17] Mayerhoefer ME Prosch H Herold CJ Weber M Karanikas G. Assessment of pulmonary melanoma metastases with 18F-FDG PET/CT: which PET-negative patients require additional tests for definitive staging? Eur Radiol. (2012) 22:2451–7. 10.1007/s00330-012-2499-x 22653282

[18] Tan GJS Berlangieri SU Lee ST Scott AM. FDG PET/CT in the liver: lesions mimicking malignancies. Abdom Imaging. (2014) 39:187–95. 10.1007/s00261-013-0043-3 24233161

[19] Chung AY Tran TB Brumund KT Weisman RA Bouvet M. Metastases to the thyroid: a review of the literature from the last decade. Thyroid. (2012) 22:258–68. 10.1089/thy.2010.0154 22313412

[20] Annunziata S Laudicella R Caobelli F Pizzuto DA Aimn Working Group Y. Clinical value of PET/CT in staging melanoma and potential new radiotracers. Curr Radiopharm. (2019) 13:6–13. 10.2174/1874471012666191015094620 31749438

[21] Cone LA Brochert A Schulz K Stone RA Kazi A Greene D. PET positive generalized lymphadenopathy and splenomegaly following interferon-alfa-2b adjuvant therapy for melanoma. Clin Nucl Med. (2007) 32:793–6. 10.1097/RLU.0b013e318148afa5 17885361

[22] Cho SY Huff DT Jeraj R Albertini MR. FDG PET/CT for assessment of immune therapy: opportunities and understanding pitfalls. Semin Nucl Med. (2020) 50:518–31. 10.1053/j.semnuclmed.2020.06.001 33059821 PMC8201415

[23] Sullivan RJ Atkins MB Kirkwood JM Agarwala SS Clark JI Ernstoff MS. An update on the Society for Immunotherapy of Cancer consensus statement on tumor immunotherapy for the treatment of cutaneous melanoma: Version 2.0. J Immunother Cancer. (2018) 6:44. 10.1186/s40425-018-0362-6 29848375 PMC5977556

[24] Wei SC Duffy CR Allison JP. Fundamental mechanisms of immune checkpoint blockade therapy. Cancer Discov. (2018) 8:1069–86. 10.1158/2159-8290.CD-18-0367 30115704

[25] Aide N Hicks RJ Le Tourneau C Lheureux S Fanti S Lopci E. FDG PET/CT for assessing tumour response to immunotherapy: report on the EANM symposium on immune modulation and recent review of the literature. Eur J Nucl Med Mol Imaging. (2019) 46:238–50. 10.1007/s00259-018-4171-4 30291373 PMC6267687

